# Sugar transporters in Fabaceae, featuring SUT MST and SWEET families of the model plant *Medicago truncatula* and the agricultural crop *Pisum sativum*

**DOI:** 10.1371/journal.pone.0223173

**Published:** 2019-09-30

**Authors:** Joan Doidy, Ugo Vidal, Rémi Lemoine

**Affiliations:** Université de Poitiers, UMR CNRS 7267, EBI "Ecologie et Biologie des Interactions", Poitiers, France; University of Alberta, CANADA

## Abstract

Sugar transporters play a crucial role for plant productivity, as they coordinate sugar fluxes from source leaf towards sink organs (seed, fruit, root) and regulate the supply of carbon resources towards the microorganisms of the rhizosphere (bacteria and fungi). Thus, sugar fluxes mediated by SUT (sucrose transporters), MST (monosaccharide transporters) and SWEET (sugar will eventually be exported transporters) families are key determinants of crop yield and shape the microbial communities living in the soil. In this work, we performed a systematic search for sugar transporters in *Fabaceae* genomes, focusing on model and agronomical plants. Here, we update the inventory of sugar transporter families mining the latest version of the *Medicago truncatula* genome and identify for the first time SUT MST and SWEET families of the agricultural crop *Pisum sativum*. The sugar transporter families of these *Fabaceae* species comprise respectively 7 MtSUT 7 PsSUT, 72 MtMST 59 PsMST and 26 MtSWEET 22 PsSWEET. Our comprehensive phylogenetic analysis sets a milestone for the scientific community, as we propose a new and simple nomenclature to correctly name SUT MST and SWEET families. Then, we searched for transcriptomic data available for our gene repertoire. We show that several clusters of homologous genes are co-expressed in different organs, suggesting that orthologous sugar transporters may have a conserved function. We focused our analysis on gene candidates that may be involved in remobilizing resources during flowering, grain filling and in allocating carbon towards roots colonized by arbuscular mycorrhizal fungi and Rhizobia. Our findings open new perspectives for agroecological applications in legume crops, as for instance improving the yield and quality of seed productions and promoting the use of symbiotic microorganisms.

## Introduction

Legumes (also called pulses) have provided a sustainable source of proteins and starch for humans and animals since the earliest of civilizations. Nowadays, legumes are internationally produced for food, feed and industrial applications [1]. They are processed for the high nutritional value of their seeds, including the production of legume flour. The recent lifestyle change with respect to meat consumption implies a reconsideration of current agricultural system to produce novel foods, such as plant proteins from grain legumes.

In agriculture, legumes are also cultivated as rotation crop, cover crop and intercropping to increase the productivity of the soil. Indeed, legumes are considered as agroecological crops, that can sustainably render nutrients available to subsequent crops without using any fertilizers [2, 3]. This is made possible through their root association with beneficial organisms naturally present in the soil biodiversity. Legumes are able to establish both symbioses with nitrogen-fixing bacteria and with mycorrhizal fungi mainly supplying phosphate [4]. As such, *Medicago truncatula* (barrel medic) became the laboratory model for studying plant-microbe interactions and *Pisum sativum* (pea) is not only a cultivated crop but also a model organism in biology since the work of famous geneticist, Gregor Mendel.

Seeds and roots thus represent two major organs for the nutritional and agroecological values of legumes. Roots and seeds are two primary carbon sinks, relying on the provision of sugars from photosynthetically source leaves. In plants, carbon fluxes are coordinated by sugar transporters, comprising sucrose transporters (SUT), monosaccharide transporters (MST) and SWEET (sugars will eventually be exported transporter). Sugar transporters from the MST, SUT and SWEET families have been shaped through natural selection for their specificity to supply carbon towards beneficial root microorganisms [5] and by human domestication to improve the nutritional values of grain crops [6].

Long-distance transport from source leaf towards sink organs is mainly mediated by SUTs, as sucrose constitutes the main carbohydrate transported through the phloem. Once sucrose reaches the release phloem, the disaccharide is exported to supply sink organs (eg seeds and roots) where it can be cleaved into monosaccharides (fructose and glucose) which are then mediated by MST. More recently, [7] identified a third family of sugar transporters, the SWEET, which seem to export both sucrose and monosaccharides. In contrast to most SUT and MST which function as active transport systems using the driving force generated by the H^+^ ATPase pump, SWEET transporters can facilitate both influx and efflux of sugars [8, 9]. Thus, SUT, MST and SWEET control carbon allocation throughout plant, directly determining crop yield, its nutritional and economical values [10]. However, complete inventory of sugar transporter gene families is only reported in a limited number of legume species.

To briefly summarize the state of the art on sugar transporter annotations, so far the best described family is the “SUT”, probably because it is a relatively small family (less than 10 genes in most species) and historically a SUT was one of the first transporters cloned in the 90’s [11]. In *Fabaceae*, SUT families were previously reported in five species (*Medicago truncatula*, *Phaseolus vulgaris*, *Glycine max*, *Cicer arietinum and Lotus japonicus* [12]). In contrast, MST represents the largest family of sugar transporter, it usually comprises more than 50 genes in higher plants. Complete MST family is only reported in three plants (Arabidopsis [13], rice [14]) including a single legume species (Medicago, [15] and updated in this paper). Regarding the third family, complete SWEET families are reported in *Fabaceae* for soybean [16] and Medicago [17]. Thus, inventories of sugar transporters families in Fabaceae genomes were only performed so far in Medicago for MtSUT ([18], genome version 3.5), MtMST ([15] genome version 3.5) and MtSWEET ([17], genome version 4.0). For pea, only partial inventories were reported for PsSUT and PsSWEET [19–21], but no PsMST has yet been published to our knowledge. Uncovering the sugar transportome in crops of agronomical interest represents an important milestone for improving the yield and quality of grain legumes.

Here, we release complete sugar transporter families (SUT MST and SWEET) of Fabaceae species, focusing primarily on the model plant *Medicago truncatula* and the agricultural crop *Pisum sativum*. We update the inventory of sugar transporter families mining the latest version of the *M*. *truncatula* genome and identify for the first time SUT MST and SWEET families of the agricultural crop *P*. *sativum*. We also screen for gene candidates involved in sugar transport towards major carbon sinks, as for instance gene clusters potentially filling nutrients into seeds and supplying carbon towards symbiotic bacteria and fungi.

## Materials and methods

### Sequence identification and retrieval of SUT, MST and SWEET families

*M*. *truncatula* (line A17) and *P*. *sativum* (cultivar Cameor) SUT MST and SWEET genes were identified by sequence similarity to previously identified families [15, 17, 18, 21] using the blastP algorithm available at the *Medicago truncatula* Genome Database (MTGD version 4.0, http://www.medicagogenome.org/ [22, 23]) and at the Pea RNA-Seq gene atlas (version 1.0, http://bios.dijon.inra.fr/FATAL/cgi/pscam.cgi, [24]). For the SUT gene families, we also mined additional *Fabaceae* species using the blastP algorithm available at Phytozome v12 (https://phytozome.jgi.doe.gov/pz/portal.html [25]) for *Glycine max* Wm82.a2 (v1), *Phaseolus vulgaris* (v2.1) and *Trifolium pratense* (v2); and the Legume Information System (https://legumeinfo.org/home, [26]) for *Arachis hypogaea*, *Arachis duranensis*, *Arachis ipaensis*, *Cajanus cajan*, *Cicer arietinum* (CDC Frontier kabuli type and ICC 4958 desi type), *Lotus japonicus* (v3.0), *Lupinus angustifolius* (v1.0), *Vicia faba*, *Vigna angularis* (v3.0), *Vigna radiata*, and *Vigna unguiculata* (v1.0). A list of all accessions presented in this work is summarized in S1 Table. A list of available Fabaceae database is also summarized in S1 Fig.

### Phylogenetic analysis of SUT, MST and SWEET families

**Fig 1:**
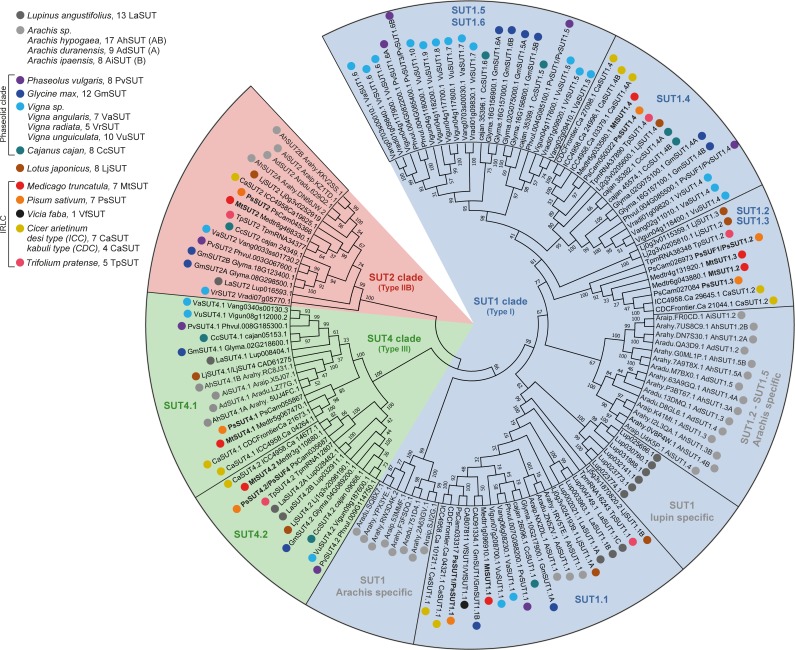
Phylogenetic tree of the Fabaceae SUT families. The 136 SUT accessions were retrieved from sixteen legume species. SUT were named upon phylogenetic grouping into particular clades (SUT1, SUT2 and SUT4). We then added a “.number” extension (eg. SUT4.1, SUT4.2) to clearly identify gene accessions within subclades. Additionally, when multiple paralogs or genes occurring from genome duplication appeared within the same clade/subclade (eg. in polyploid species like soybean and *Arachis hypogaea*), we added a capital letter extension to clearly identify all representative accessions (eg. GmSUT2A et GmSUT2B). Several accessions belonging to the basal species lupin and Arachis SUT1 subclades (SUT1.1 lupin specific and SUT1 Arachis specific subclades) were not annotated/named due to limited genome information within Genistoid and Dalbergioid species (see S1 Fig).

**Fig 2:**
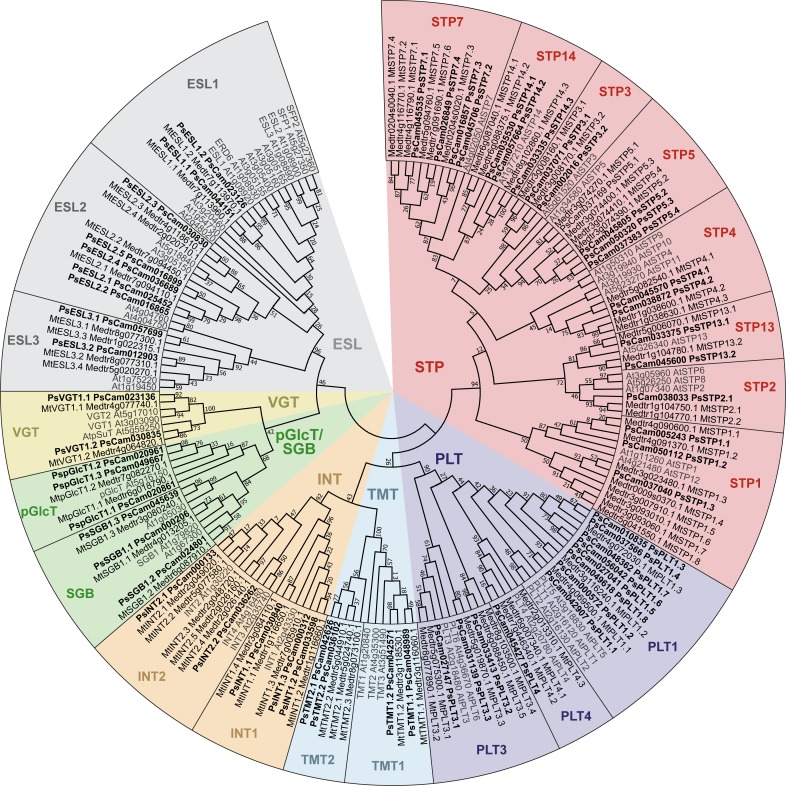
Phylogenetic tree of Medicago, pea and Arabidopsis MST families. The 72 MtMST (regular font) were retrieved from the *M*. *truncatula* genome v4.0, 59 PsMST (bold font) from the pea Gene Atlas and 53 AtMST (grey font) from the Arabidopsis genome v11. MtMST and PsMST were named upon phylogenetic grouping into the seven MST clades: sugar transport protein (STP), polyol/monosaccharide transporter (PLT), inositol transporter (INT), vacuolar glucose transporter (VGT), tonoplast membrane transporter (TMT), pGlcT/SGB for plastidic glucose transporter (pGlcT) and suppressor of G Protein Beta1 (SGB1) and early-responsive to dehydration six-like (ESL). We then added a “.number” extension (eg. STP1.1 to STP1.8) to clearly identify gene accessions within subclades.

**Fig 3:**
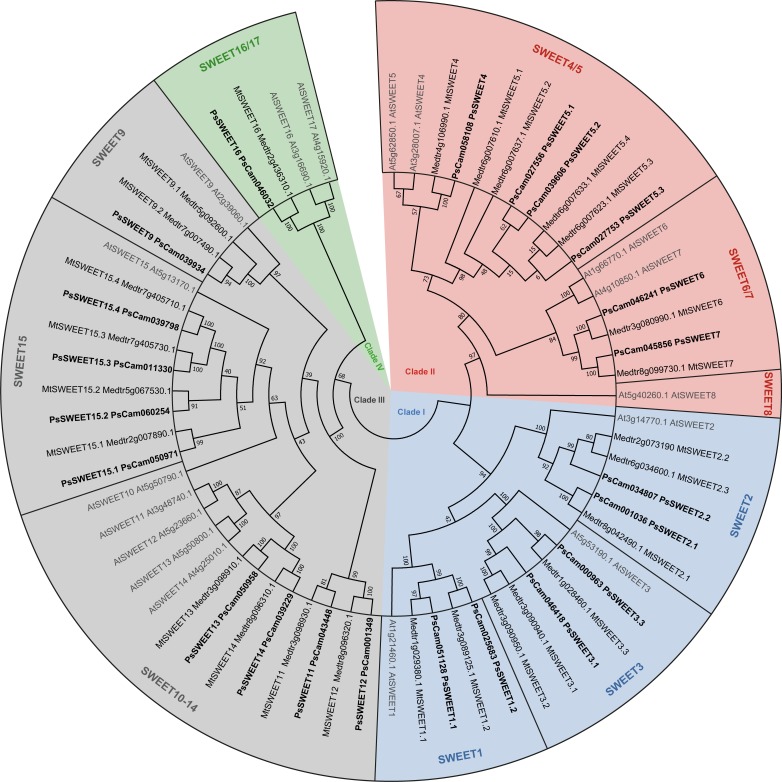
Phylogenetic tree of Medicago, pea and Arabidopsis SWEET families. The 26 MtSWEET (regular font) were retrieved from the *M*. *truncatula* genome v4.0, 22 PsSWEET (bold font) from the pea Gene Atlas and 17 AtSWEET (grey font) from the Arabidopsis genome v11. PsSWEET were named upon phylogenetic grouping into the four SWEET clades: clade I (SWEET1, SWEET2 and SWEET3 subclades), clade II (SWEET4/5 and SWEET5/6 subclades). clade III (SWEET9, SWEET11/12, SWEET13/14, SWEET15 subclades) and clade IV (SWEET16). We then added a “.number” extension (eg. SWEET1.1 and SWEET1.2) to clearly identify gene accessions.

After accession retrieval, only sequences showing a full-length coding sequence were retained for alignment. Alignment of amino acid sequences of SUT MST and SWEET families was performed using the ClustalW Multiple alignment algorithm on BioEdit [27] and then exported to MEGA7 software [28] for construction of the SUT MST and SWEET phylogenetic trees presented in Figs 1, 2 and 3 respectively. The evolutionary history was inferred by using the Maximum Likelihood method based on the JTT matrix-based model [29]. Initial tree for the heuristic search were obtained automatically by applying Neighbor-Join and BioNJ algorithms to a matrix of pairwise distances estimated using a JTT model, and then selecting the topology with superior log likelihood value. All positions containing gaps and missing data were eliminated.

### Gene expression analysis

**Fig 4:**
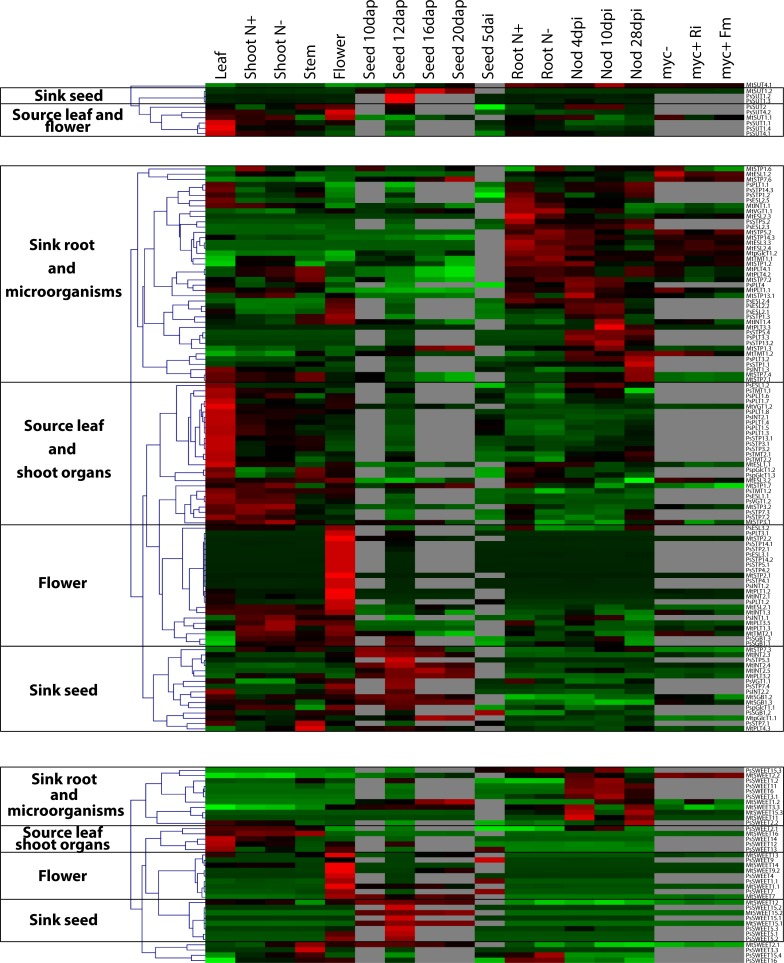
Gene expression patterns of SUT, MST and SWEET. Transcriptomics profile of Medicago and pea SUT (top panel), MST (middle panel) and SWEET (bottom panel) from plant samples (leaf, shoot, stem, flower, seed and root); shoot and root supplemented with nitrogen (N+) or nitrogen limited (N-); roots inoculated with beneficial microorganisms like Rhizobia (at 4, 10, 28 days post inoculation), arbuscular mycorrhizal fungi (*Rhizophagus irregularis*: myc+Ri or *Funneliformis mosseae*: myc+Fm) and non-mycorrhizal root control (myc-). The heat map shows z-score normalized expression for genes/rows (green: low expression, red: high expression) and a hierarchical gene tree, highlighting coexpression clusters.

All expression data, presented in Fig 4, were retrieved from the *Medicago truncatula* Gene Expression Atlas (MtGEA, microarray data available at https://mtgea.noble.org/v3/, [30]) and the Pea RNA-Seq gene atlas [24]. The experiments displayed on the heat maps were selected as follow: for *M*. *truncatula*, gene expression in different organs (leaf, stem flower, seed, nodules; [31]), shoot and root supplemented with nitrate (NO_3_^-^: N+) or nitrogen limited (N-; [32]), mycorrhizal (roots inoculated with *Rhizophagus irregularis* or *Funneliformis mosseae*) and non-mycorrhizal root systems [33]; for *P*. *sativum*, all experiments were retrieved from the Pea RNA-Seq gene atlas [24]. The heat map was created using Multiple Experiment Viewer (Mev) software [34] with expression normalization by z-score for genes/rows to correct the color display between rows. Gene trees were generated using the hierarchical clustering (HCL) function in Mev with Pearson correlation and average linkage clustering to identify co-expression clusters from carbon source and sink organs.

## Results and discussion

First, we mined sugar transporters in Fabaceae genomes and propose a new nomenclature for correctly naming sugar transporters (SUT MST and SWEET). For instance, we named SUT1 sucrose transporters belonging to clade 1. We then added a “.number” extension (eg. SUT1.1, SUT1.2) when multiple paralogous genes were present within the same clade. Finally, we tried our best to correctly identify orthologous genes from different species by employing identical extensions (eg. MtSUT1.1, PsSUT1.1), when such members clearly appeared within the same branch of a particular clade. We also named other families (MST and SWEET) using the same nomenclature. In the future, we propose that all sugar transporters to be named as following (see Figs 1, 2 and 3). A list of all sugar transporters presented in this work, along with the naming history, is available as supplementary data (S1 Table).

We raise the attention of the scientific community to consistently annotate sugar transporter according to their phylogenetic position, rather than naming transporters following the chronological order of their discovery. We believe that this new nomenclature will enable consistent mapping and annotation of sugar transporters across plant genomes. We also believe that all SUT, MST and SWEET identified in this study will soon be mapped in Fabaceae genomes, including in the upcoming v5 of the Medicago genome and in the reference pea genome, just released this September [35].

### The sucrose transporter (SUT) families in Fabaceae genomes

Our phylogenetic analysis confirms that all SUT accessions fall into the dicotyledonous specific clade (SUT1/Type I) and the dicotyledonous-monocotyledonous clades (SUT2/Type IIA, SUT4/Type III), but none belong to the monocotyledonous specific clade (SUT2/Type IIB; [12]). The Dicots SUT family is clearly divided in three clades (SUT1, SUT2 and SUT4, [12, 36]), here we named MtSUT and PsSUT according to this clade convention. The SUT1 clade comprises the highest number of accessions with four SUT1 members, the SUT2 clade shows a single accession and the SUT4 clade comprises a gene duplicate for both Medicago and pea (S2 Fig).

Here, we present SUT families comprising 136 accessions from 16 *Fabaceae* species (Fig 1), including newly identified families from *Lupinus angustifolius* (12 LaSUT), *Arachis sp*. (17 AhSUT, 9 AdSUT, 8 AiSUT), *Vigna sp*. (7 VaSUT, 5 VrSUT and 10 VuSUT), *Cajanus cajan* (8 CcSUT), *Lotus japonicus* (8 LjSUT) and *Trifolium pratense* (5 TpSUT). Complete SUT family in Fabaceae genomes had previously been uncovered from *Medicago truncatula* (7 MtSUT), *Glycine max* (12 GmSUT), *Lotus japonicus* (8 LjSUT), *Phaseolus vulgaris* (8 PvSUT) and *Cicer arietinum* (7 CaSUT) ([12, 18]; number of gene accessions reviewed and updated in this paper). Partial SUT family in *P*. *sativum* were also uncovered from RNA-seq data (7 PsSUT) ([21]; number of gene accessions reviewed and updated in this paper). Noteworthy, single SUT accessions had also been characterized before the release of genome and transcriptome in the 90’s and 2000’s from several legume species (S1 Table), historically in soybean [37, 38], *Vicia faba* [39], *P*. *sativum* [19, 20, 40], *Lotus japonicus* [41, 42] and *Phaseolus vulgaris* [19].

#### The SUT1.1 subclade comprises a single protein involved in phloem loading in Dicots

Within the SUT1 clade, the SUT1.1 subclade comprises a single accession in most species (Fig 1), which is so far the best characterized SUT members. This SUT1.1 protein is responsible of sucrose phloem loading in apoplasmic loading species [43]. Therefore, *SUT1*.*1* gene mutation causes sugar accumulation, reduced photosynthesis and chlorotic lesions in source leaves as well as reduced growth of sink organs and thereby an overall stunted plant growth [44]. However, no *SUT1*.*1* mutant has yet been generated in a legume species, which seem to load sucrose in the phloem via the apoplast [19, 45]. As such, *MtSUT1*.*1* and *PsSUT*.*1* show a high expression profile in source leaves and are co-expressed in different organs (Fig 4), suggesting that orthologous SUT1.1 may have a conserved function for loading the phloem.

Within the SUT1.1 subclade, we find a single accession for Phaseolid and IRLC species, which seems to originate from genes both present in the basal Papilionideae species (LaSUT1.1A, B or C, AiSUT1.1, AdSUT1.1 and AhSUT1.1; Fig 1 and S1 Fig). Besides, a SUT1 lupin specific subclade diverges at the basis of the SUT1.1 subclade and comprises 6 gene paralogs. Also, a sister SUT1 clade is specific to *Arachis* species with numerous gene paralogs. These SUT1 specific subclades were not annotated/named due to limited information within this basal Genistoid and Dalbergioid species.

#### Other SUT1 subclades comprise multiple paralogous genes

The SUT1.2/SUT1.3 subclade comprises two gene paralogs in most species and is only represented by species from the IRLC and Robinioid clades (S1 Fig). This subclade comprises sucrose facilitator (SUF, [19]). SUF seems to transport sucrose both in and out of the cell, according to the concentration gradient. Thus, such SUF weakly complement mutant yeast and their transport kinetics are not affected by ATPase inhibitors [18, 19]. In parallel to SWEET sucrose exporters (see clade III SWEET, Fig 3), such facilitator systems may also be involved in the export of sucrose towards carbon sinks.

The SUT1.4 subclade comprises a single gene locus in most species of the Phaseolid, IRLC and Robinioid clades. Transporters from this clade also seem to facilitate sucrose transport at the plasma membrane, but only a single member of this subclade has been functionally characterized so far (PvSUF1 here reannotated as PvSUT1.4, [19]).

Noteworthy, MtSUT1.4 (Medtr6g033580) was not annotated in the 3.5 version of the *M*. *truncatula* genome. Therefore, the MtSUT family was previously reported to comprise only six genes (including three in the SUT1 clade [18]). Here, we update the number of SUT loci in *M*. *truncatula* genome, the MtSUT family now comprises seven genes (MtSUT1.4 being the fourth accession in the SUT1 clade).

The SUT1.5/SUT1.6 subclade comprised two gene duplicates in most diploid species and is only represented by species belonging to the Phaseolid clade. Surprisingly, this subclade contains several accessions only in *Vigna unguiculata*, which may be an assembly or annotation issue from the 1.0 version of this genome. So far, a single protein from this subclade has been characterized (PvSUT1 here reannotated as PvSUT1.5, [19]). PvSUT1.5 does not seem to facilitate sucrose transport, but rather seems to actively transport sucrose against its concentration gradient using an active H^+^ATPase transport system. Interestingly, *PvSUT1-5* and *PvSUT1-6* showed their highest expression profiles in flower and seed coat [19].

#### The SUT2 clade, a single accession with unclear kinetics and function

The SUT2 clade comprise a single gene locus in most Monocots and Dicots plants [18], this clade cannot further be divided into subclades (Fig 1). In polyploid species, multiple SUT2 loci can be observed, each copy corresponding to the locus from the parental diploid genome. For instance, this can be reported for the tetraploid genomes of soybean (GmSUT2A and GmSUT2B) and *Arachis hypogaea* (AhSUT2A and AhSUT2B).

First described to act as sucrose sensors [46], since SUT2 proteins were not functional when expressed in yeast. *In planta*, SUT2 mutant lines are affected in fruit yield, seed development [47] and in symbiotic interaction with mycorrhizal fungi [48], suggesting that SUT2 imports sucrose back inside plant cells to control sugar supply towards carbon sinks. In addition, SUT2 seems to interact with components of the phytohormonal signals, also suggesting its role as a “transceptor” (both a transporter and a sensor, [48]). Here, *PsSUT2* shows a low and constant expression level in most tissues of *P*. *sativum* and similar SUT2 expression pattern is observed in other plant species (S3 Fig). The physiological roles of SUT2 remain unclear, more works need to be performed to unravel their exact kinetics of transport and/or sensing functions.

#### Fabaceae SUT4 clade contains two accessions for remobilizing vacuolar reserve

The SUT4 clade comprises two gene accessions, divided in two subclades (SUT4.1 and SUT4.2). This is the case for most (if not all) Fabaceae species presented here. Apparently, this characteristic is not limited to legume species, as many other land plants (switchgrass, *Panicum virgatum*; cassava, *Manihot esculenta*; *Brassica rapa*; apple, *Malus domestica*; cotton *Gossypium raimondii*) including basal Monocots (banana, *Musa acuminate* and date palm, *Phoenix dactylifera*), also comprise two SUT4 accessions [12].

Interestingly, the polyploid soybean species also possess two SUT4 accessions, GmSUT4.1 and GmSUT4.2 (and not four accessions originating from the diploid ancestral genomes). In contrast, the other polyploid species *A*. *hypogea* possess two gene copies in the SUT4.1 subclade originating from the diploid parents (AhSUT4.1A and AhSUT4.1B), but no SUT4.2 is present in the genome of all *Arachis* species. The second basal Fabaceae species lupin (Fig 1), possesses both a SUT4.1 and a SUT4.2 accession. A comprehensive study of basal land plant genomes may unfold the evolution of the SUT4 clade.

Functionally, members of the SUT4.1 clade have been studied in detail. Indeed, SUT4.1 functions as active transport system coupled to H^+^ATPase, exporting intracellular sucrose reserves out of the vacuole [49]. In contrast, only a single protein from the SUT4.2 has been characterized as a sucrose facilitator “SUF” (PsSUF4, [19]), mediating sucrose transport (in and out). Although SUT4.1 proteins have clearly been shown to be addressed to the tonoplast [49], the exact localization of SUT4.2 members have not yet been shown. The presence of dileucine motifs, sufficient for vacuolar targeting in both SUT4.1 and SUT4.2 sequences, seems to comfort the hypothesis of the vacuolar localization of all SUT4 proteins [50].

### The monosaccharide transporter (MST) families in Medicago and pea

As previous classification of *Fabaceae* MST had not yet been performed, we decided to add Arabidopsis (AtMST) sequences in our phylogenetic analysis (Fig 2). Indeed, Arabidopsis is the only plant species where the MST family is clearly annotated in seven clades [13, 36]. We suggest that the convention for naming MST clades to be reviewed in the future, as the clade nomenclature is currently ambiguous. Indeed, clades are either named according to substrate specificity [sugar transport protein (STP), polyol/monosaccharide transporter (PLT), inositol transporter (INT)], subcellular localization [vacuolar glucose transporter (VGT), tonoplast membrane transporter (TMT), plastidic glucose transporter (pGlcT)], mutant-recovering phenotype [suppressor of G Protein Beta1 (SGB1)] or even a stress condition that induced gene expression [early-responsive to dehydration six-like (ESL)]. For instance, a consortium of experts may agree on a reference classification, as it was performed for the NRT1/PTR family [51].

That said, in this second part we mined MST from Medicago and pea. We decided to annotate MtMST and PsMST based on the current clade convention, using the name of the representative gene orthologs previously annotated in Arabidopsis. Here, we report 72 MtMST and 59 PsMST, aligned with 53 AtMST (Fig 2). Noticeably, the PsMST family comprise 18% less accessions than in the Medicago genome. In contrast to the SUT family (S2 Fig), which shows similar number of accessions and seems fully identified in both species, we envision that the PsMST family may not be completely uncovered. For instance, we previously had identified 58 MtMST in the 3.5 version of the Medicago genome [15], here our second search in the 4.0 version enabled the identification of additional MtMST. Moreover, the number of predicted transmembrane domains seems to confirm that the newly identified transporters are full length transporters (S1 Table) since they possess a comparable number of transmembrane domains than Arabidopsis homologs, which have been functionally characterized.

We also confirm that MtMST and PsMST accessions fall into the seven MST clades (Fig 2), described above. Thus, we identified 30 MtSTP and 21 PsSTP divided in eight subclades (STP1, STP2, STP3, STP4, STP5, STP7, STP13, STP14); 11MtPLT and 12 PsPLT divided in three subclades (PLT1, PLT3 and PLT4); 5 MtTMT and 4 PsTMT divided in two subclades (TMT1 and TMT2); 9 MtINT and 5 PsINT divided in two subclades (INT1 and INT2); 5 MtpGlcT/SGB and 6 PspGlcT/SGB divided in pGlcT and SGB subclades; 2 MtVGT and 2 PsVGT in a single VGT clade; finally, 10 MtESL and 9 PsESL divided in three subclades (ESL1, ESL2 and ESL3).

Based on the current number of accessions in Arabidopsis and Medicago genomes, we compared MST family expansion within each clade (S2 Table). We observed a total of 19 additional MST genes in Medicago genome compared to Arabidopsis. The gene duplications are primarily observed in STP, PLT and INT, as these clades seems greatly expanded in Fabaceae genomes, whereas the ESL clade shows an expansion in Arabidopsis genome. Interestingly, similar observations were also made for the rice STP, PLT and ESL clades [14] but not for INT. Functionally, these great MST expansions in plant genomes have not yet been investigated in detail.

To our knowledge, only one monosaccharide transporter has been characterized in *M*. *truncatula*. Mtst1 (a gene paralog of MtSTP1.2 identified in a different subpopulation of *M*. *truncatula*) is a glucose transporter present at the plasma membrane of root cells colonized by arbuscular mycorrhizal fungi [52]. Here, we also retrieved both *MtSTP1*.*2* and *PsSTP1*.*2* orthologs in the gene cluster expressed in roots in response to symbiotic microorganisms (Fig 4).

### The SWEET families in Medicago and pea

Complete inventory of the SWEET family has already been performed in the latest version (4.0) of the *M*. *truncatula* genome, uncovering 26 MtSWEET [17]. Here, we decided to take advantage of this annotation to correctly name the 22 PsSWEET orthologous accessions in pea (Fig 3). Complete SWEET family was also identified from a third Fabaceae species, the tetraploid soybean comprises 52 GmSWEET [16], corresponding approximatively to twice the number of accessions than the diploid species here.

We show that MtSWEET and PsSWEET accessions fall into the four SWEET clades, previously uncovered in Arabidopsis [7]. Arabidopsis only possesses 17 AtSWEET; gene expansion in Fabaceae genomes seems to have mainly occur through duplications of SWEET1, SWEET2, SWEET3 within clade I, SWEET5 within clade II and SWEET15 within clade III (S2 Table).

SWEET transporters have been discovered recently [7], it is well established that plant SWEETs are divided in four clades (clade I, II, III and IV), but subclades have never been defined. Here, we show that Clade I can be further divided in three subclades (SWEET1, SWEET2 and SWEET3 subclades; Fig 3). Clade I transporters seems to transport sugar across plasma membranes. Recently, MtSWEET1b (renamed here MtSWEET1.2) was shown to control glucose supply towards arbuscular mycorrhizal fungi [53]. Another leguminous clade I SWEET, LjSWEET3 was shown to mediate sucrose transport [54] towards nodules. Therefore, clade I SWEET show a broader substrate spectrum than SWEET from other clades and seem involved in sugar partitioning towards symbiotic organisms (see 4.4).

Clade II is divided into two subclades (SWEET4/5 and SWEET6/7 subclades; Fig 3), as Fabaceae do not seem to comprise a SWEET8 subclade, in contrast to Arabidopsis (S2 Table). Most clade II SWEET were shown to be involved in glucose export towards reproductive organs (pollen [55] and seeds [6]) and fungal pathogens [7], but no clade II members has yet been studied in a legume species.

Clade III members are further divided in three subclades (SWEET9, SWEET10-14 and SWEET15 subclades; Fig 3). Interestingly, Fabaceae do not seem to possess a SWEET10 subclade, in contrast to Arabidopsis (S2 Table). Within clade III, the SWEET15 subclade shows a great expansion with four gene paralogs for both Medicago and pea. The single AtSWEET15 member in Arabidopsis was shown to transport sucrose and seems located at both the plasma membrane and Golgi apparatus [56]. The role of the multiple SWEET15 members in Fabaceae has not yet been investigated. Nevertheless, clade III SWEET are the most studied so far. Namely, SWEET11 and SWEET12 where shown to be involved in loading sucrose towards phloem vessels in Arabidopsis [57]. In Fabaceae, SWEET11 members may have a different function or may not be direct orthologs of AtSWEET11 (see paragraph below). As the only clade III SWEET studied so far in a fabacean species MtSWEET11, specifically transports sucrose towards root nodules ([17], Fig 4).

Noteworthy our phylogenetic analysis highlights that SWEET4/5 (clade II) and SWEET10-14 (clade III) subclades may not yet be fully resolved, as some tree branches show low bootstrap value (Fig 3). Interestingly, both subclades could not be completely resolved through a more comprehensive analysis using a wide range of plant species, including basal plants [8].

Within clade IV, Medicago and pea only possesses a single gene copy, named SWEET16 (Fig 3). In contrast, Arabidopsis shows two genomic loci (AtSWEET16 and AtSWEET17; S2 Table). Clade IV members were shown to transport sugars (notably fructose) across vacuolar membranes [58, 59]. While *MtSWEET16* seems majorly expressed in leaf, *PsSWEET16* is both expressed in shoot and root (Fig 4). The functional roles of this unique SWEET16 in Fabaceae remain to be studied.

To conclude, in Fabaceae three SWEET have been studied in detail. LjSWEET3, MtSWEET1.2 and MtSWEET11 seems involved in sugar partitioning towards symbiotic soil microorganisms [17, 53, 54]. Our following analysis aims to identify transporter candidates exploiting transcriptomic data from model and agronomic legumes.

### Gene expression patterns of the sugar transportome

Here, the transportome is defined by the range of genes that encodes proteins contributing to transport sugars across cellular membranes in Medicago and pea genomes (Figs 1, 2 and 3). We analyze the expression patterns of the sugar transportome, mining transcriptomic data and selecting experimental conditions of interest (Fig 4).

Our heat map mainly shows a typical transcriptional activation pattern (“switch on”), suggesting that transporters are induced in organs where they exhibit a physiological function (Fig 4 and S4 Fig). Such pattern indicate that sugar transporters are primarily regulated through transcriptional activation of their gene expression [60]. For instance, *SUT1*.*1* expression is switched on in shoots and leaves, where its protein product actively transports sucrose towards phloem sieve tubes. In contrast *SUT1*.*1* expression is lower in root system where its role remains unknown (Fig 4). Supporting this hypothesis, it was proposed that phloem loading capacity is directly proportional to the rate of transcription of symporter genes in source leaves [60]. Noteworthy, sugar transporters also show posttranslational regulations, as for instance through protein oligomerization and phosphorylation [61, 62].

#### Gene cluster induced in source leaves

We noticed that sugar transporters with a high expression in shoot organs are usually switched off in root system (and vice versa; Fig 4). First, we identify SUT MST and SWEET gene clusters induced in source leaf and shoot organs, relevant to their physiological functions.

In the cluster of genes co-expressed in source leaf, we find major transporters involved in long distance transport of sucrose. We also show that many Medicago and pea gene orthologs are often located within the same cluster. For instance, see MtSUT1.1 and PsSUT1.1 positioned within the same cluster, both genes are putatively involved in loading sucrose towards phloem (see 1.1). We also identify PsSWEET12 in this cluster, its ortholog in Arabidopsis (AtSWEET12) was shown to be responsible for the efflux of sucrose into the apoplasm, thus feeding sucrose for further import by SUT1.1 into the sieve element-companion cell [57]. Alongside SWEET12, several clade III SWEET were also recovered within this sub-cluster (*MtSWEET13*, *PsSWEET13* and *PsSWEET14*), these candidates may also participate in long distance transport of sucrose from source to sink organs.

Interestingly, we also identify a large cluster of MST co-expressed in source leaf. This cluster comprises several MST paralogs positioned within the same sub-cluster, specifically expressed in leaf (see PsPLT1, PsTMT2, PsSTP3). A transporter from the PLT1 clade was characterized in *L*. *japonicus*, LjPLT4 is a xylitol transporter and shows its maximum of expression in leaf [63]. In source organs, MST are required at the plastid membranes for the export fixed carbon, at the tonoplast for intracellular reserve and at the plasma membrane to recover carbohydrate generated from the cell wall and to retrieve carbohydrates leaking into the apoplast [64].

#### Gene cluster induced in flowers

Another cluster clearly stands out in flower with several MST and SWEET specifically expressed during floral development (Fig 4). Three SWEET orthologs are co-expressed in this “flower cluster” (*MtSWEET1*.*1*, *PsSWEET1*.*1*, *MtSWEET7*, *PsSWEET7*, *MtSWEET9*.*2*, *PsSWEET9*), here SWEET seems to coordinate sugar allocation during flowering (Fig 4). Consistent with our findings, the SWEET9 ortholog in Arabidopsis is a sucrose transporter involved in nectar production [65] and seems specifically expressed in flowers (S4 Fig). In parallel, *AtSWEET1* and *AtSWEET7* are also highly expressed during floral development [9], but we could only verify this information for *AtSWEET1* based on available transcriptomic data (S4 Fig). The role of SWEET1 and SWEET7 in flower has not yet been deciphered.

A large cluster of MST transport systems is also switched on in flowers (Fig 4). In the literature, only VGT members (AtVGT1 and AtpSuT) were clearly shown to transport both monosaccharide and sucrose necessary for flowering onset [66, 67]. Here, our transcriptomic analysis identifies different MST induced in Fabaceae flowers (Fig 4), indicating that Fabaceae species might employ a different cascade of transporters during floral development.

#### Gene cluster induced in sink seeds

We also screened for gene candidates involved in sugar transport towards major carbon sinks, as for instance gene clusters potentially filling nutrients into seeds. Here again, we uncover orthologous genes switched on in both pea and Medicago, namely SWEET15 from clade III (*MtSWEET15*.*1*, *PsSWEET15*.*1* and *MtSWEET15*.*2*, *PsSWEET15*.*2*; Figs 3 and 4). The unique AtSWEET15 gene in Arabidopsis is mostly expressed during seed development (S4 Fig). SWEET15 are sucrose transporters involved in nutrient provision towards the embryo and for seed filling in soybean, Arabidopsis and rice [56, 68, 69]. The orthologous genes in soybean (*GmSWEET15*.*1* and *GmSWEET15*.*2*) are essential for embryo development by mediating sucrose export from the endosperm to the embryo during early in seed development [69]. Moreover, several paralogous pea accessions of the SWEET4/5 clade II were also identified in this “sink seed cluster” (*PsSWEET5*.*1*, *PsSWEET5*.*2* and *PsSWEET5*.*3*, Figs 3 and 4); it was shown that ZmSWEET4 and OsSWEET4 mediate seed filling in cereal grain crops [6]. Noteworthy, it has been shown that SWEET4 genes, controlling the agronomically important trait of filling nutrients into seeds, were subjected to selection during crop domestication [6]. Here we identify large clusters of SWEET and MST candidates induced in Medicago and pea seeds (Fig 4). However, the exact function of these sugar transporters has not yet been investigated in a leguminous grain crop.

#### Gene cluster induced by root symbioses

As sugars are also diverted toward non-plant sinks, we screened for candidates involved in sugar transport towards beneficial microbes living in the rhizosphere. Indeed, plant colonization by heterotrophic organisms increases sugar demand, consequently long-distance transport mediated by SUT is also impacted. We previously showed that MtSUTs show a fine-tuning regulation in response to symbiotic partners [5, 18]. Here we only have limited data regarding MtSUT regulation in response to AMF (arbuscular mycorrhizal fungi), however all PsSUT and MtSUT (except *MtSUT1*.*2*, *PsSUT1*.*2* and *PsSUT1*.*3*) show a fine-tuning regulation during rhizobial development (Fig 4).

We also retrieve putative vacuolar sucrose transporters MtTMT1.1 and MtTMT1.3 (Fig 4) belonging to the MST family (Fig 2). Confirming the robustness of our candidate search, *MtTMT1*.*1* was previously identified in neighboring cells colonized by AMF (arbuscular mycorrhizal fungi; [70]).

Within the MST family, we recover several transporters from the STP clades expressed during root symbioses (Fig 4). As previously mentioned (see 2), MtSTP1.2 is the only MST characterized in Medicago and was shown to be induced in cells colonized by AMF and in neighboring root cells [52]. Also, we retrieved several STP5 accessions in this root clusters, namely orthologous genes *MtSTP5*.*2*, *PsSTP5*.*2* as well as *PsSTP5*.*4*. We further identify transporters from the STP13 subclade, both *MtSTP13*.*1* and *PsSTP13*.*2* seem induced in root nodule (Fig 4). Interestingly, STP13 proteins seem to regulate carbon diversion towards both symbiotic and parasitic microorganisms [71, 72]. STP transporters compete with fungal and bacterial transporters to retrieve carbohydrates back towards plant cells [73].

Our analysis also confirmed that SWEET3 homologs (*PsSWEET3*.*1*, *MtSWEET3*.*3* and *LjSWEET3* [54]), SWEET11 orthologs (*MtSWEET11* and *PsSWEET11* [17]) and SWEET15 orthologs (*MtSWEET15*.*3* and *PsSWEET15*.*3* [74]) are specifically induced in root nodules across Fabaceae species (Fig 4).

Interestingly, numerous transporters are strongly activated by mycorrhizal symbioses, but none seem AM specific [18, 75]. For instance, we identify SWEET1 orthologs (*MtSWEET1*.*2* and *PsSWEET1*.*2*; Fig 4). *MtSWEET1*.*2* is induced in both nodulated and mycorrhizal roots and the transporter localizes to the plant-fungal interface [53, 76].

Thus, we uncover transporters regulated in response to both N-fixing bacteria and mycorrhizal fungi. Early signaling pathway resulting in a successful symbiotic interaction is shared between mycorrhiza and Rhizobia, this common symbiotic pathway is well described (for review see [77]). In contrast, the occurrence of a “common symbiotic transport pathway”, shared by both fungi and bacteria in the later stages of the symbioses and regulating the nutrient trades with the plant, remains an open question.

## Conclusions

Thus, we release sugar transporter families in Fabaceae species, focusing on the model plant *Medicago truncatula* and the agricultural crop *Pisum sativum*. We identify SUT MST and SWEET candidates controlling sugar allocation within plants and between organisms. Identifying candidates driving carbon cycles towards the plant-microbe-soil continuum may lead towards agroecological applications, as for instance improving the yield and quality of grain productions by promoting the use of symbiotic microorganisms naturally present in the soil biodiversity. The appropriate management of these ecosystem services will impact on natural resources with an obvious net gain for human society [78].

## Supporting information

S1 Fig Phylogenetic tree of the Papilionoideae subfamily. The tree is a simplified representation with triangles describing the major clades, including the plastid DNA inverted repeat-lacking clade (IRLC). Adapted from [79] and The Legume Phylogeny Working Group 2013 [80].
(PDF)

S2 Fig Phylogenetic tree of Medicago and pea SUT families. The 7 MtSUT (regular font) were retrieved from the *M*. *truncatula* genome v4.0 and 7 PsSUT (bold font) from the pea Gene Atlas.
(PDF)

S3 Fig SUT2 gene expression patterns. *Pisum sativum* expression was retrieved from the pea gene atlas, *Lotus japonicus* from ExpAt (https://lotus.au.dk/expat/), *Glycine max*, Arabidopsis and *Zea mays* from ePlant (https://bar.utoronto.ca/eplant/).
(PDF)

S4 Fig Gene expression patterns of Arabidopsis sugar transporters. Expression profiles of AtSUT (top panel), AtMST (middle panel) and AtSWEET (bottom panel) from plant samples (rosette leaf #6, vegetative rosette, stem second internode, flower stage 15, seed stage 3, 5, 7 and 9, and roots; [81]). The heat map shows z-score normalized expression by root mean square (green: low expression, red: high expression).
(PDF)

S1 Table List of SUT MST and SWEET accessions presented in this work. (XLSX)

S2 Table Comparative analysis of MST and SWEET clades in *Arabidopsis*, *Medicago truncatula* and *Pisum sativum*. (XLSX)
