# Supplementary material for: Sugar transporters in Fabaceae, featuring SUT MST and SWEET families of the model plant Medicago truncatula and the agricultural crop Pisum sativum
Source: PLoS One. 2019 Sep 30;14(9):e0223173. doi: 10.1371/journal.pone.0223173 (PMC6768477; doi:10.1371/journal.pone.0223173)
Supplement: S1 Fig — The tree is a simplified representation with triangles describing the major clades, including the plastid DNA inverted repeat-lacking clade (IRLC). Adapted from [79] and The Legume Phylogeny Working Group 2013 [80]. (PDF) [file pone.0223173.s001.pdf]

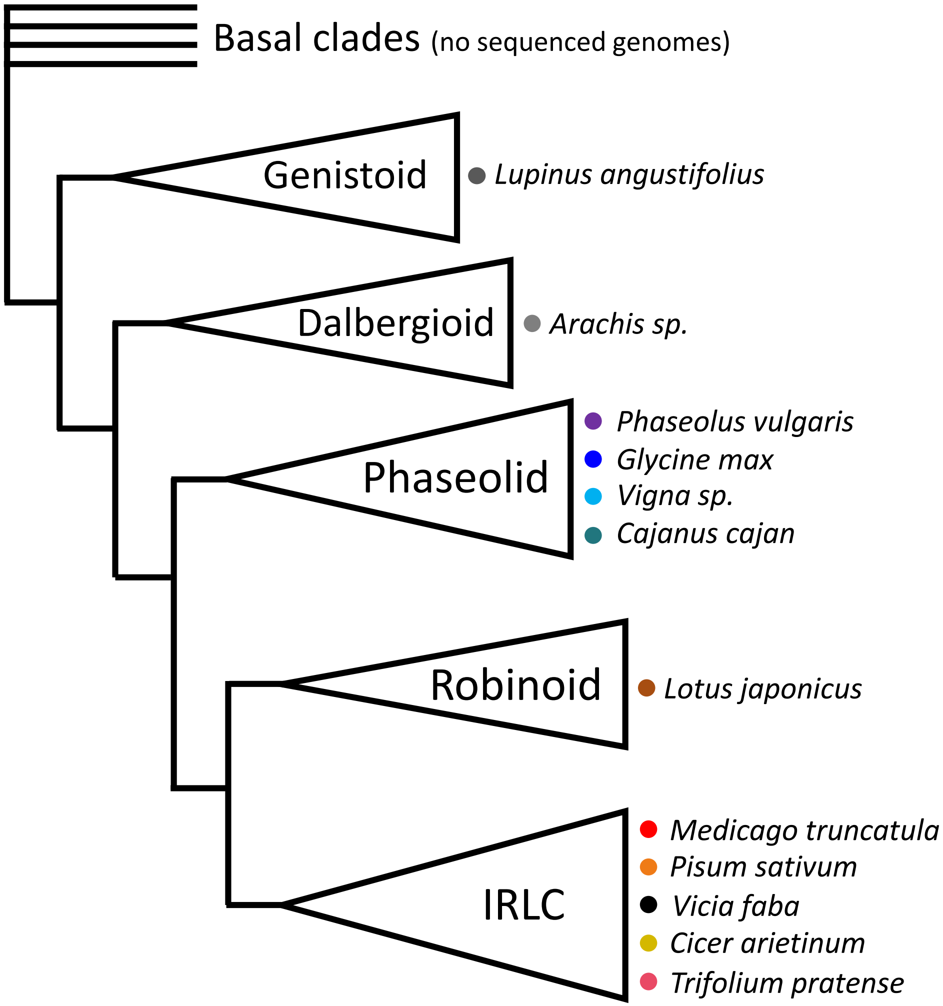

S1 Fig. Phylogenetic tree of the Papilionoideae subfamily. The tree is a simplified representation with triangles describing the major clades, including the plastid DNA inverted repeat-lacking clade (IRLC). Adapted from Chantreuil et al. 2016 (https://doi.org/10.1093/dnares/dsw020) and The Legume Phylogeny Working Group 2013 (http://dx.doi.org/10.5167/uzh-78167).
