# Supplementary material for: Sugar transporters in Fabaceae, featuring SUT MST and SWEET families of the model plant Medicago truncatula and the agricultural crop Pisum sativum
Source: PLoS One. 2019 Sep 30;14(9):e0223173. doi: 10.1371/journal.pone.0223173 (PMC6768477; doi:10.1371/journal.pone.0223173)
Supplement: S2 Fig — The 7 MtSUT (regular font) were retrieved from the M. truncatula genome v4.0 and 7 PsSUT (bold font) from the pea Gene Atlas. (PDF) [file pone.0223173.s002.pdf]

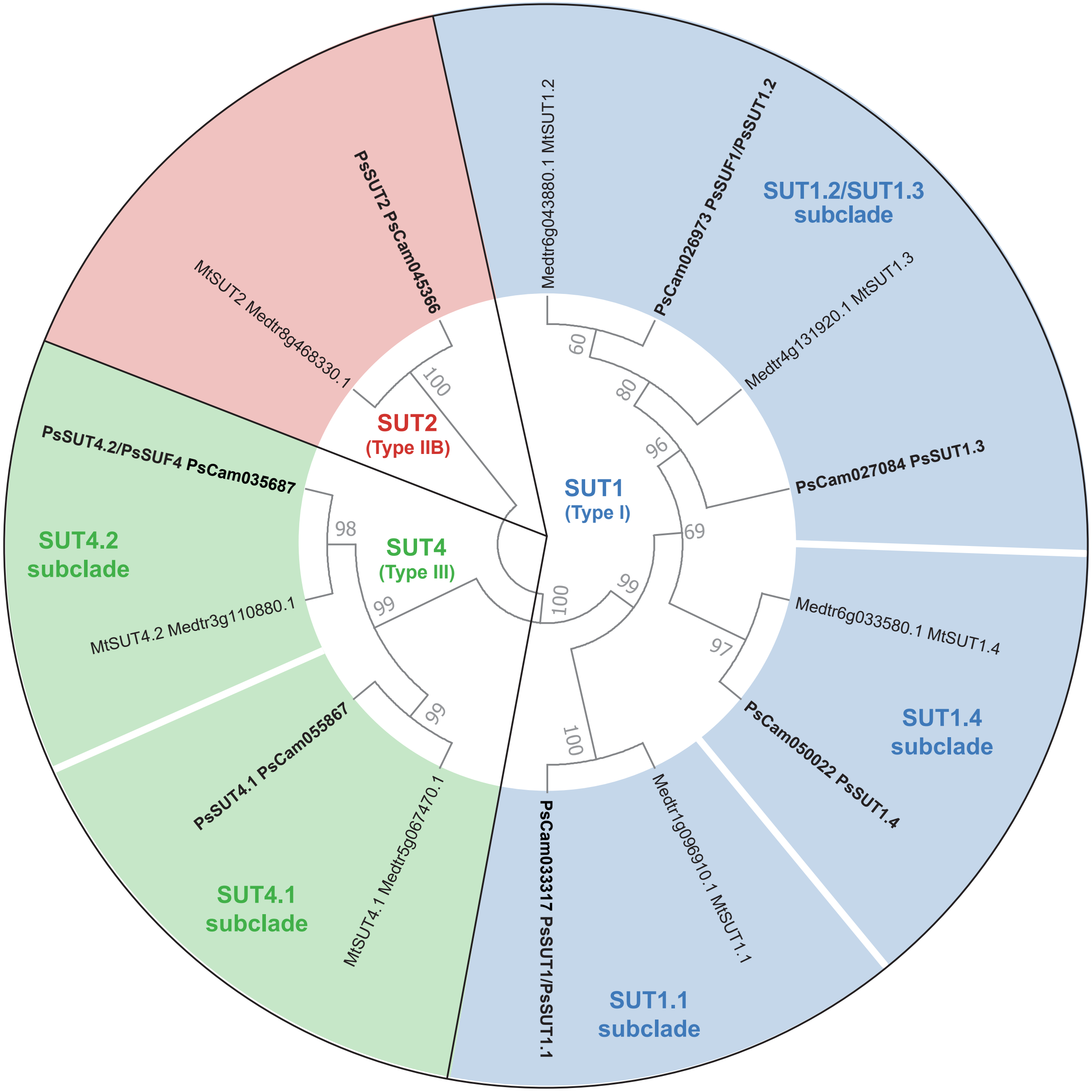

S2 Fig : Phylogenetic tree of Medicago and pea SUT families.
The 7 MtSUT (regular font) were retrieved from the M. truncatula genome v4.0 and
7 PsSUT (bold font) from the pea Gene Atlas.
