# Supplementary material for: Sugar transporters in Fabaceae, featuring SUT MST and SWEET families of the model plant Medicago truncatula and the agricultural crop Pisum sativum
Source: PLoS One. 2019 Sep 30;14(9):e0223173. doi: 10.1371/journal.pone.0223173 (PMC6768477; doi:10.1371/journal.pone.0223173)
Supplement: S3 Fig — Pisum sativum expression was retrieved from the pea gene atlas, Lotus japonicus from ExpAt (https://lotus.au.dk/expat/), Glycine max, Arabidopsis and Zea mays from ePlant (https://bar.utoronto.ca/eplant/). (PDF) [file pone.0223173.s003.pdf]

PsSUT2 PsCam045366

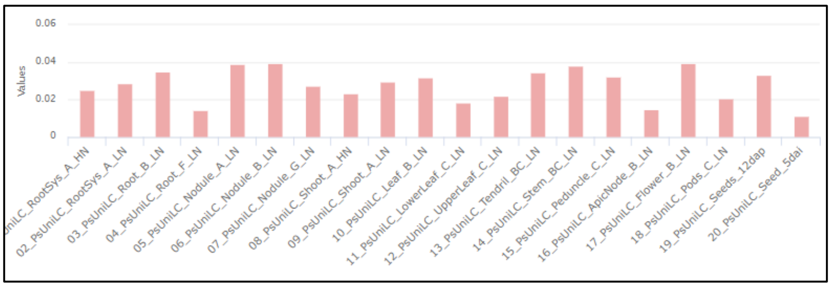

LjSUT2 Lj0g3v0252919

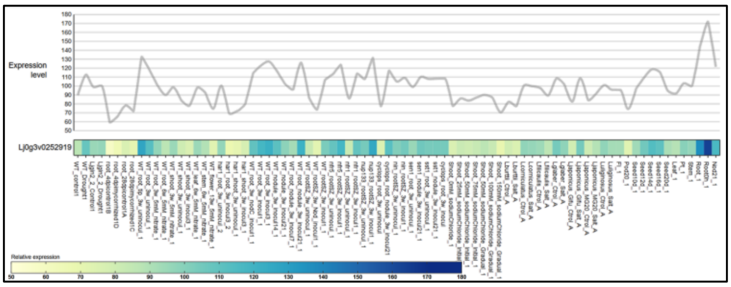

GmSUT2A Glyma.08G298500

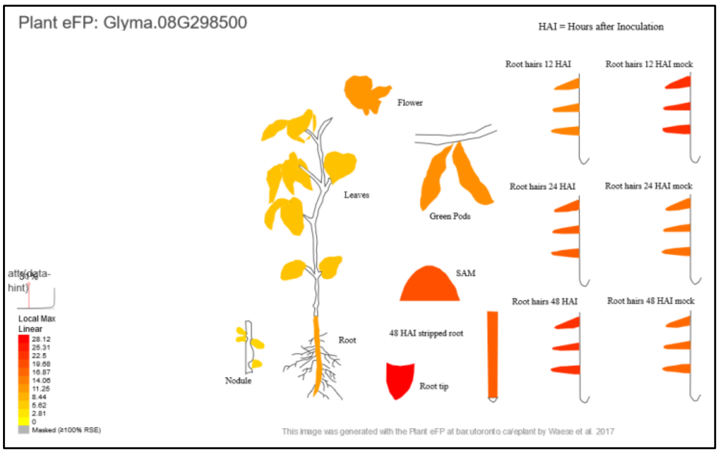

GmSUT2B Glyma.18G123400

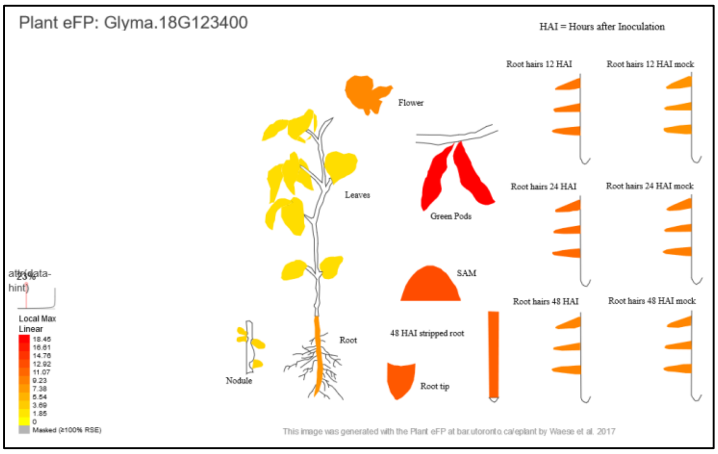

AtSUC3/AtSUT2 At2g02860

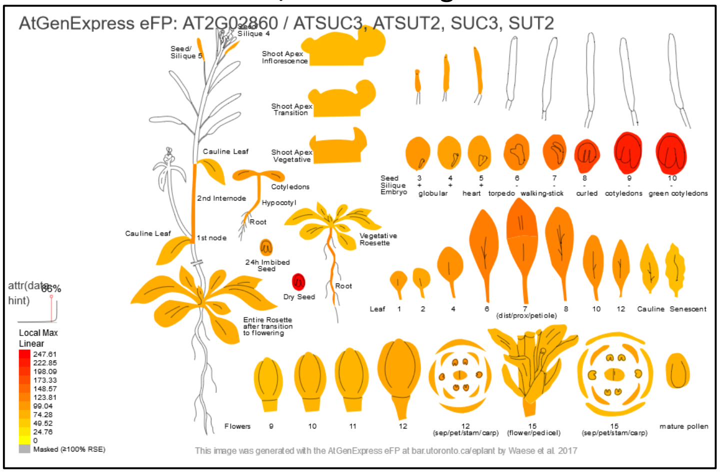

ZmSUT2 GRMZM2G145107

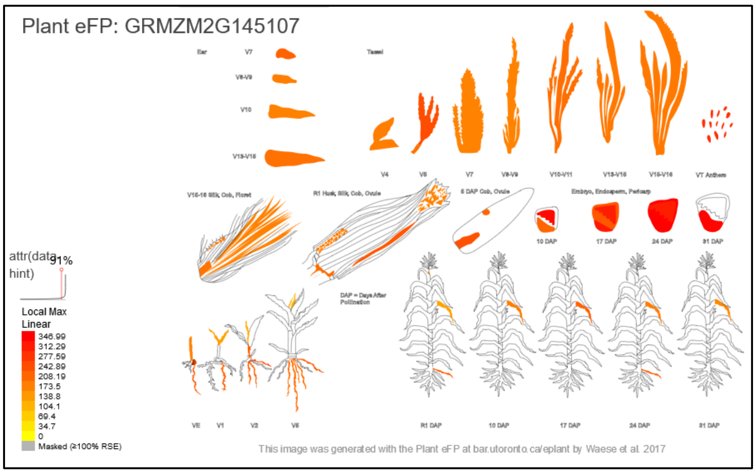

S3 Fig: SUT2 gene expression patterns. *Pisum sativum* expression was retrieved from the pea gene atlas, *Lotus japonicus* from ExpAt (https://lotus.au.dk/expat/), *Glycine max*, Arabidopsis and *Zea mays* from ePlant (https://bar.utoronto.ca/eplant/).
