# Supplementary material for: Sugar transporters in Fabaceae, featuring SUT MST and SWEET families of the model plant Medicago truncatula and the agricultural crop Pisum sativum
Source: PLoS One. 2019 Sep 30;14(9):e0223173. doi: 10.1371/journal.pone.0223173 (PMC6768477; doi:10.1371/journal.pone.0223173)
Supplement: S4 Fig — Expression profiles of AtSUT (top panel), AtMST (middle panel) and AtSWEET (bottom panel) from plant samples (rosette leaf #6, vegetative rosette, stem second internode, flower stage 15, seed stage 3, 5, 7 and 9, and roots; [81]). The heat map shows z-score normalized expression by root mean square (green: low expression, red: high expression). (PDF) [file pone.0223173.s004.pdf]

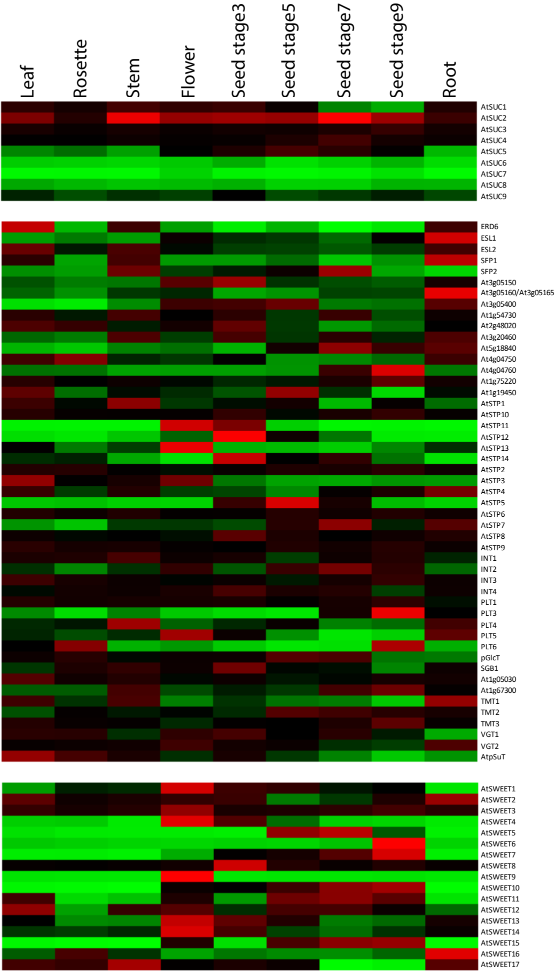

S4 Fig: Gene expression patterns of Arabidopsis sugar transporters. Expression profiles of AtSUT (top panel), AtMST (middle panel) and AtSWEET (bottom panel) from plant samples (rosette leaf #6, vegetative rosette, stem second internode, flower stage 15, seed stage 3, 5, 7 and 9, and roots ; [81]). The heat map shows z-score normalized expression by root mean square (green: low expression, red: high expression).
